# Serum Ionized Calcium May Be Related to White Matter Lesion Volumes in Older Adults: A Pilot Study

**DOI:** 10.3390/nu5062192

**Published:** 2013-06-18

**Authors:** Martha E. Payne, Cortnee W. Pierce, Douglas R. McQuoid, David C. Steffens, John J. B. Anderson

**Affiliations:** 1 Department of Psychiatry and Behavioral Sciences, Duke University, Durham, NC 27710, USA; E-Mails: cortnee.pierce@duke.edu (C.W.P.); douglas.mcquoid@duke.edu (D.R.M.); 2 Neuropsychiatric Imaging Research Laboratory, Duke University, Durham, NC 27705, USA; 3 Department of Psychiatry, University of Connecticut Health Center, Farmington, CT 06030, USA; E-Mail: steffens@uchc.edu; 4 Department of Nutrition, Gillings School of Global Public Health, University of North Carolina, Chapel Hill, NC 27599, USA; E-Mail: jjb_anderson@unc.edu

**Keywords:** calcium, serum ionic calcium, white matter lesion, white matter hyperintensity, brain, older adult, depression

## Abstract

White matter lesions have detrimental effects upon older adults, while serum calcium levels have been associated with elevated vascular risk and may be associated with these lesions. Depression, a serious mental disorder characterized by disturbances in calcium metabolism, may be an important contributor to any calcium-lesion relationship. This cross-sectional pilot study examined the association between serum ionized calcium (the physiologically active form of calcium) and white matter lesion volumes in a sample of depressed and non-depressed older adults (*N* = 42; 60 years and older). Serum ionized calcium was determined using an ion-selective electrode technique, while lesion volumes were estimated from magnetic resonance imaging using an automated expectation-maximization segmentation. A linear regression model, controlling for age and group (depression *vs.* comparison), showed a trend for a positive relationship between serum ionized calcium and white matter lesion volume (β = 4.34, SE = 2.27, *t* = 1.91, *p* = 0.063). Subsample analyses with depressed participants showed a significant positive relationship between higher ionic calcium and greater lesion volume (β = 6.41, SE = 2.53, *t* = 2.53, *p* = 0.018), but no association was found for non-depressed participants. Sex-specific subsample analyses showed a significant positive relationship between higher calcium and greater lesion volume in men only (β = 7.49, SE = 3.42, *t* = 2.19, *p* = 0.041). These preliminary results indicate that serum ionized calcium may be associated with white matter lesions in older adults, particularly among men and individuals with depression. Larger studies are needed to confirm these findings.

## 1. Introduction

**Figure 1:**
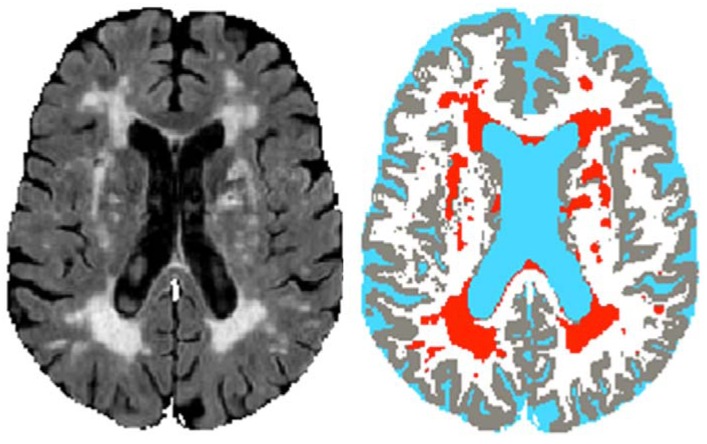
White matter lesions (WMLs) shown on magnetic resonance imaging: Fluid-attenuated inversion recovery image (left), tissue classification image (right; lesions in red). Images are for illustrative purposes only. The images were derived from a 3 Tesla MRI acquisition and processing for a participant in the parent study (NCODE), although this individual was not a participant of the present serum calcium study.

Brain white matter lesions (WMLs), also known as white matter hyperintensities or leukoaraiosis, are areas of damage viewed on magnetic resonance imaging (MRI) (Figure 1). WMLs, which are primarily ischemic in origin [1], are common in older adults and promote the development and progression of late-life depression [2,3], a debilitating condition with a prevalence of between 2.7% and 10.1% in elders [4,5]. WMLs also increase risk of other detrimental health conditions, including cognitive decline, dementia, stroke, physical disability, hip fracture and death [6,7,8,9,10,11,12,13]. Aging of the population, combined with the obesity epidemic and related hypertension, may significantly increase prevalence of WMLs and their outcomes.

Serum calcium is one unexplored factor that may influence WMLs, especially given that WMLs result primarily from ischemic damage [1]. Elevated serum calcium levels, even those within the normal range, have been linked to greater risk of death, myocardial infarction, other cardiovascular events, and stroke [14,15,16,17]. In addition, greater baseline serum calcium has been associated with diminished performance as well as faster decline in cognitive testing in older adults [18]. One mechanism by which serum calcium may promote WMLs is arterial calcification. Elevated serum calcium has been associated with aortic and carotid calcification and plaques [19,20,21], while arterial calcification has been cross-sectionally and longitudinally associated with greater WMLs [22,23,24]. Determining the role of serum calcium for WMLs is critical because of the damaging effects of these lesions and the potentially modifiable nature of serum calcium. This question is especially important for women’s health because women are at greater risk of depression and dementia, conditions that are partially promoted by WMLs [2,8]. In addition, women are more likely to use calcium supplements, products which may cause greater perturbations in serum calcium than do food forms of calcium [25,26].

Several conditions have been linked to disturbances in calcium metabolism, including hypertension [27], renal disease [28], and, of particular relevance for the present study, depression. A serious and common mental illness, depression is a leading cause of disability worldwide [29]. Depression has been characterized as a disorder of calcium metabolism as evidenced by elevations in serum [30] and cerebrospinal [31] calcium levels, and alterations in intracellular calcium [32]. In addition, dietary calcium has been shown to promote learned helplessness [33], an animal model of depression, while calcium channel blockers alleviate learned helplessness [34] and may also augment antidepressant therapy in humans [35]. This calcium dysregulation may be an unexplored explanatory factor for the elevation of WMLs observed with late-life depression.

The aim of this pilot study of older adults was to evaluate the relationship between WMLs and serum ionized calcium (serum Ca_i_). We hypothesized that higher levels of serum Ca_i_ would be positively associated with greater WML volumes. A secondary aim was to explore the role of calcium in depressed participants, given that depressed individuals exhibit calcium dysregulation and have higher WML volumes. In addition, we sought to explore the potential for sex differences in any identified calcium-WML relationship.

## 2. Methods

This pilot study was conducted as part of a larger longitudinal clinical examination of depressed and non-depressed older adults (NeuroCognitive Outcomes of Depression in the Elderly [NCODE]), which began in 1994 and had ongoing enrollment until 2011 [36]. Serum samples, for calcium and other measures, were collected between 6/1/2011 and 6/30/2012 from newly-enrolled participants as well as previously-enrolled participants when they came in for an annual study visit. MRI brain scans were acquired on all study participants, beginning at their baseline visit. For this project only participants with 3 Tesla (3T) MRIs (acquired 2003 and later) were included; the MRI closest in time to lab draw was used for WML quantification.

### 2.1. Sample

All participants who had both serum Ca_i_ and WML data (from 3T MRI) were included in the current study. This sample included patients who met DSM-IV diagnostic criteria for major depressive disorder at study baseline, and never-depressed comparison participants recruited from the community. Participants were aged 60 years or older, and could speak and write English. 

Exclusion criteria included a concurrent diagnosis of a psychiatric or neurological illness, and significant cognitive impairment (as indicated by a Mini-Mental State Examination score of less than 24 out of 30) [37]. Comparison participants (controls) were required to have no evidence of a current or lifetime depression diagnosis.

After complete description of the study to the participants, written informed consent was obtained. A separate consent form was completed by previously-enrolled participants for the serum Ca_i_ and other assessments. New participants completed one consent form that included lab assessments in addition to other study procedures. This research protocol has been reviewed and approved by the Duke University Institutional Review Board (IRB), and was in compliance with Health Insurance Portability and Accountability Act (HIPAA) guidelines.

### 2.2. Treatment

Depression patients received individualized treatment from a psychiatrist, who followed them throughout the study. Most depressed participants received antidepressant medication.

### 2.3. Measures

#### 2.3.1. Laboratory Assessment

Lab measures included serum Ca_i_, parathyroid hormone (PTH), 25-hydroxy-vitamin D, and phosphorus. The majority of participants were fasting for the blood draw (*N* = 37), although this was not a requirement for any lab measure except phosphorus. After collection and centrifuge, if indicated, samples were taken on the same day via LabCorp courier to a LabCorp facility (Burlington, NC) for analysis. Serum Ca_i_ utilized a separate gel-barrier tube (kept at room temperature, and labeled “Do Not Open”), and assessment was conducted with an ion-selective electrode methodology (reference interval: 4.5–5.6 mg/dL). Plasma PTH utilized an EDTA tube (kept refrigerated), and assessment was conducted by electrochemiluminescence immunoassay (reference interval: 15–65 pg/mL). Vitamin D measurement utilized a gel-barrier tube (kept refrigerated) and immunochemiluminometric assay (reference interval: 32–100 ng/mL). Serum phosphorus utilized a gel-barrier tube (kept at room temperature), and was calculated by calorimetric procedure (reference interval: 2.5–4.5 mg/dL).

#### 2.3.2. White Matter Lesion (WML) Volumes

Cranial MRI was performed using the 8-channel parallel imaging head coil on a 3T whole-body MRI system (Trio, Siemens Medical Systems, Malvern, PA, USA). Proton density (PD), T1-weighted, T2-weighted, and fluid-attenuated inversion recovery (FLAIR) images were acquired. Parallel imaging was employed with an acceleration factor of 2. The pulse sequence parameters have been described previously [38]. The MR images were processed for WML volumes using an automated method in the Neuropsychiatric Imaging Research Laboratory (NIRL). This automated 4-channel lesion segmentation, which takes advantage of FLAIR images for WML detection, was performed, as previously developed by our group [38]. The method was optimized for vascular WML assessment in elderly subjects. WMLs are detected as “outliers” to the normal tissue distributions. The method is capable of distinguishing and classifying WMLs and other brain tissues simultaneously.

#### 2.3.3. Covariates

Covariates presented include basic demographics (age, sex, race), years of education, group (depression *vs.* comparison), and hypertension. Age was that at time of MRI scan. Hypertension (“high blood pressure”) was included with self-reported physical health items from the National Institute of Mental Health (NIMH) Diagnostic Interview Schedule [39] portion of the Duke Depression Evaluation Schedule (DDES), which was administered annually. MMSE, a global measure of cognition, was assessed every 6 to 12 months. For hypertension and MMSE, the data obtained from the closest assessment (to lab collection date) was used.

### 2.4. Statistical Analyses

All statistical analyses were done using JMP software, version 8.0 (SAS Institute, Inc.; Cary, NC, USA). Significance was defined at *p* < 0.05 level, although trends (0.05 ≥ *p* < 0.10) were considered given the small sample size. Given small numbers of minority participants, race was dichotomized as White *vs.* non-White.

For bivariate analysis, *t*-tests were performed on continuous level variables and Chi-Square tests were performed on categorical variables testing for differences between depressed and non-depressed comparison participants. A simple regression model was fit to examine relationship between Ca_i_ and WML volume. A multivariable model was fit to examine this relationship after inclusion of covariates. Given the limited sample size, covariates were restricted to group (depressed *vs.* non-depressed) and age only. Hypertension was considered as a covariate, but was not included because of missing values (data only available for *N* = 35 (of *N* = 42)). Secondary analyses included an examination of separate regression models for each participant group (depression and comparison), as well as men *vs.* women.

## 3. Results

### 3.1. Participant Characteristics

This pilot sample of older adults included a total of 42 participants (*N* = 29 depressed participants, *N* = 13 non-depressed comparison participants). See Table 1 for participant characteristics, including comparison of depressed and non-depressed groups. Depressed individuals were more like to report hypertension (χ^2^ = 8.15, *df* = 1, *p* = 0.0043) than non-depressed participants. There were no other group differences. Of note, higher WML volumes were shown to be significantly related to lower MMSE scores (β = −0.13, SE = 0.05, *t* = −2.46, *p* = 0.019).

**Table 1:** Participant characteristics: Sociodemographics, hypertension, lab values, and white matter lesions (WMLs) ^a^.

	Total (*n* = 42)	Depressed (*n* = 29)	Non-depressed (*n* = 13)	*p* value ^b^
Age in years	68.4 (6.7)	68.3 (6.5)	68.8 (7.5)	0.8
Sex (female)	19 (45%)	15 (52%)	4 (31%)	0.2
Race (White)	39 (93%)	26 (90%)	13 (100%)	0.1
Education in years	15.8 (1.6)	15.8 (1.7)	16.0 (1.5)	0.7
Hypertension (yes) ^c^	9 (26%)	9 (38%)	0 (0%)	0.004
Mini-Mental State Examination (total score)	28.7 (1.3)	28.8 (1.1)	28.5 (1.7)	0.5
Serum ionized calcium (mg/dL)	5.1 (0.2)	5.1 (0.2)	5.1 (0.2)	0.5
25-hydroxy-vitamin D (ng/mL)	34.5 (12.9)	35.2 (14.7)	32.9 (7.8)	0.6
Phosphorus (mg/dL)	3.5 (0.5)	3.5 (0.4)	3.5 (0.6)	0.7
Parathyroid hormone (pg/mL)	31.5 (14.2)	31.8 (13.4)	30.8 (17.7)	0.9
WML volume (mL)	5.4 (3.6)	5.4 (3.3)	5.3 (4.2)	0.9

^a^ mean (SD) or # (%). ^b^* p* value for difference between groups (chi-squared test used to compare proportions; *t*-test used to compare means). ^c^* N* = 35.

### 3.2. Serum Ionized Calcium (Serum Cai) and White Matter Lesion (WML) Volume

#### 3.2.1. Main Model

**Figure 2:**
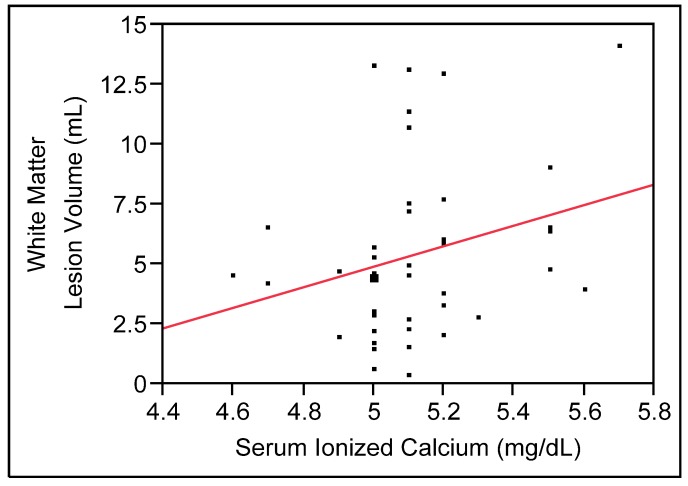
A positive relationship between serum ionized calcium (Ca_i_) and white matter lesions (WMLs) (*N* = 42; trend in multivariable model, *p* = 0.06).

To examine the effect of serum Ca_i_ on WML volume, a simple regression model was run. This model showed a trend for a positive relationship between serum Ca_i_ and WML volume (β = 4.30, SE = 2.40, *t* = 1.79, *p* = 0.080; see Figure 2). To examine the effect of serum Ca_i_ on WML volume while controlling for potential confounders, a multivariable linear regression model was run which included covariates. Given the small sample, only age and group (depression *vs.* comparison) were included as covariates. This multivariable model also showed a trend for a positive relationship between serum Ca_i_ and WML volume (β = 4.34, SE = 2.27, *t* = 1.91, *p* = 0.063).

#### 3.2.2. Secondary Analyses

**Figure 3:**
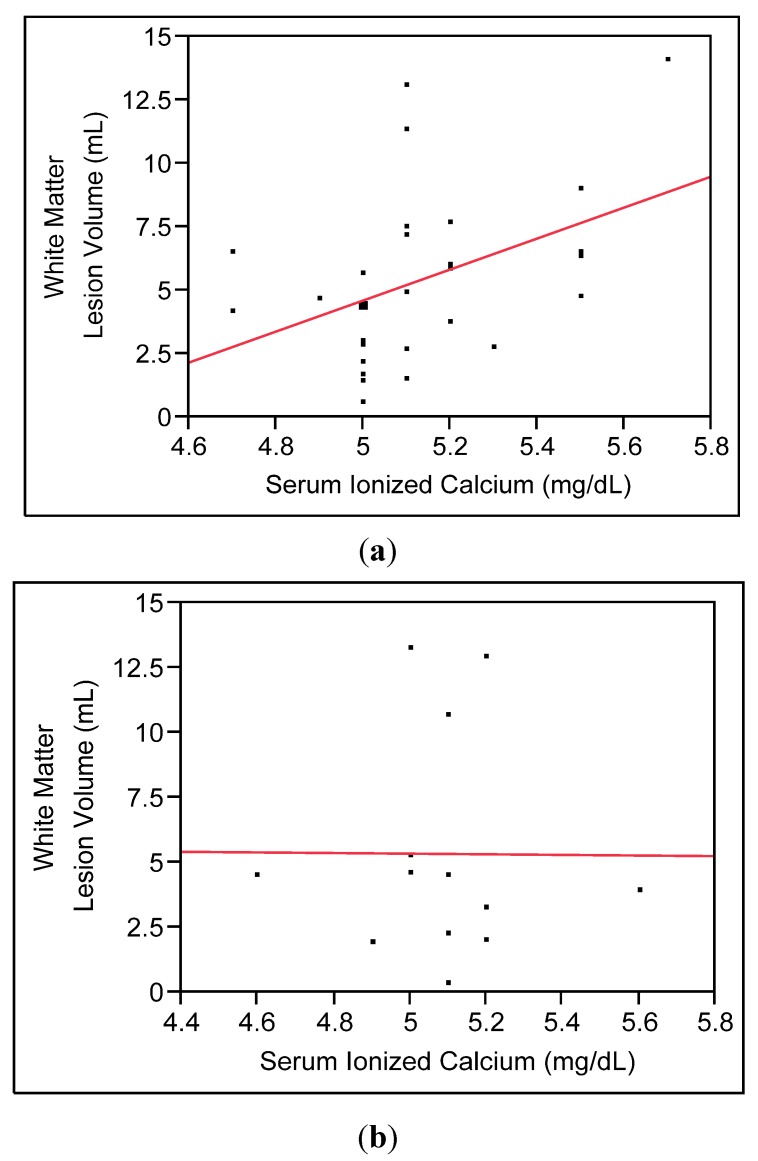
Bivariate relationships between serum ionized calcium (Ca_i_) and white matter lesions (WMLs) in depressed group, (**a**) significant in multivariable model, *p* = 0.02) and non-depressed comparison group; (**b**) not significant in multivariable model, *p* = 0.3).

Role of Depression. As shown in Table 1, there were no significant differences between depressed and comparison participants on either serum Ca_i_ or WMLs. However, given that depression has been shown in prior studies to be related to both of these measures, separate models for depressed and non-depressed groups were examined (see Figure 3). Depressed participants showed a significant positive relationship between higher serum Ca_i_ and greater WML volume, after controlling for age (β = 6.41, SE = 2.53, *t* = 2.53, *p* = 0.018), while there was no significant association found in non-depressed comparison participants (β = −4.04, SE = 3.45, *t* = −1.17, *p* = 0.27).

Male-Female Differences. There were no significant differences between men and women on either serum Ca_i_ or WMLs. However, separate models for men and women, controlling for age and group (depression *vs*. comparison), examined the potential for sex differences, and showed a significant positive relationship between higher serum Ca_i_ and greater WML volume in men (β = 7.49, SE = 3.42, *t* = 2.19, *p* = 0.041), while there was no significant association found in women (β = 1.85, SE = 3.25, *t* = 0.57, *p* = 0.58).

## 4. Discussion

This pilot study showed a trend for a positive relationship between greater serum Ca_i_ and WMLs, after controlling for age, in older adults who participated in a study of late-life depression. In subsample analyses, there was a significant positive relationship between serum Ca_i_ and WMLs in depressed participants, while there was no significant relationship in non-depressed participants. Similarly, men exhibited a significant serum Ca_i_-WML relationship, while women did not. Although preliminary, these findings suggest that serum Ca_i_ may be associated WMLs in older adults, and this relationship may differ by sex and in those with depression. Further studies are needed to replicate these pilot findings.

A positive relationship between serum Ca_i_ and WMLs is consistent with prior work that has identified linkages between WMLs and both dietary calcium and arterial calcification [22,23,24,40], as well as the detrimental effects of elevated serum calcium levels upon arterial calcification [19,20,21]. Our prior study of older adults showed that greater calcium intakes were associated with higher volumes of brain lesions, indicating calcium’s potential as an contributing factor [40], but ionic calcium measurements were not available at that time. Since WMLs result primarily from ischemia, this calcium-lesion finding is consistent with recent studies, including one done with the Heidelberg cohort (*N* = 23,980), showing that greater calcium intakes, especially those from calcium-containing dietary supplements, may elevate one’s risk of cardiovascular outcomes [15,17,41,42,43,44]. Supplemental calcium may increase the risk of WMLs because of its bolus-like effects upon serum calcium concentrations [25,26]. Furthermore, elevated serum calcium, even within the normal range, has been associated with arterial calcification [19,21,45]. Calcification of vascular smooth muscle occurs with aging, renal disease, diabetes, and atherosclerosis, and has been associated cross-sectionally and longitudinally with WMLs [23,46,47,48,49,50,51]. Initially viewed as simply an indicator of atherosclerotic burden, evidence now suggests that calcification of coronary and carotid arteries are independently associated with WMLs [23,24,47,48,49,50,52]. Two longitudinal cohorts (Zoetermeer and Rotterdam) found that midlife aortic calcification predicted WMLs in later adulthood [22]. In addition to effects upon arterial calcification, serum calcium may have direct influence on brain health, as shown by calcium’s effects on neurotransmitter turnover and neurotoxic mechanisms [53,54,55]. Thus, serum calcium may be an important mediator or contributor to WMLs via either arterial calcification or another mechanism.

The finding of a significant Ca_i_-WML relationship in depressed participants, while not present in the non-depressed subsample, may be an anomaly reflecting the variability in this small sample, or alternatively may indicate that depression, a disorder characterized by calcium dysregulation, has an important influence on serum calcium and brain relationships. The notion that depression may be a moderator of the calcium-WML relationship is not new. In the Rotterdam Study both coronary and aortic calcification were strongly associated with depressive disorders (odds ratio = 3.89) [56]. In addition, a small study of elderly depressives and comparison subjects found that carotid atherosclerosis was highly correlated (*r* = 0.55) with WMLs but only in those with depression [57], suggesting that depression is a key moderator. In addition to depression, other disorders of calcium metabolism, including hypertension and renal disease, may be important to a calcium-WML relationship. In the current study, it is possible that the apparent influence of depression was due in part to hypertension, as these two clinical entities overlapped in this sample (as found in other studies).

The sex difference observed in the Ca_i_-WML relationship, which was significant in men but not women, may relate to sex differences in serum calcium during the lifespan [14] and the effects of menopause in women [58], or it may result from differences in underlying cardiovascular disease. However, as with other findings for this study, sample size is a major limitation. An analysis of variance examined the concurrent influence of sex and depression (with interactions) and found neither to be significant. It would be premature to speculate further on the implications of the pilot findings until they are replicated with a larger sample.

This study has strengths which include the use of an automated method for quantifying WML volumes from MRI [38]; this method provides substantial advantages over technician-influenced semi-automated quantitative methods as well as over qualitative rating scales for WMLs, which often have poor reliability. In addition, this study examined serum ionized calcium (serum Ca_i_) rather than total calcium. Serum Ca_i_ is a superior measure of true calcium status because it is the physiologically active component of blood calcium, it is tightly regulated by calcium binding hormones, and it is not influenced by protein content of serum.

Limitations of this pilot study include its cross-sectional nature, which precludes the establishment of a directional relationship between serum Ca_i_ and WMLs. The small sample size is also a concern, limiting both power and our ability to control for potential confounders, including hypertension and medication use, as multiple variables could not be included in the models. Generalizability to older population groups is limited because the study sample was drawn from a clinical psychiatric study of subjects age 60 and over.

## 5. Conclusions

This pilot study showed that higher levels of serum ionized calcium (serum Ca_i_) may be positively associated with brain white matter lesions (WMLs) in older adults, particularly among men and in those with depression. These preliminary findings need to be replicated in larger samples. No change in calcium recommendations is warranted at the present time, at least none based upon this pilot, cross sectional study. These preliminary results may indicate that WMLs are another adverse outcome associated with elevated serum calcium levels, and point to the potential importance of screening for serum ionic calcium in the older adult population.
